# 

**Published:** 1914-05

**Authors:** 


### The Woman's Department

CONDUCTED BY MRS. F. E. DANIEL

COLLABORATORS

Dr. J. S. Abbott, Dairy and Food Commissioner of Texas; Miss Eleanor Brackenridge, President of the Equal Franchise Society, San Antonio; Dr. G. Henri Bogart, Associate Editor, Utah Medical Journal, Paris, Ill.; Mrs. Isabella Charles Davis, Second Vice President National Order of "King's Daughters," New Jersey; Mrs. Jno. S. Turner, Recording Secretary, Texas Congress of Mothers; Dr. Marvin B. Grace, Seguin; Dr. Margaret Holliday, Women's Physician, University of Texas, Austin; Dr. John S. Turner, President State Medical Association; Dr. Ralph Steiner, State Health Officer; Dr. L. H. Sackett, Instructor in the Philosophy of Education, University of Texas; Dr. F. S. White, Supt. S. W. Texas Lunatic Asylum, San Antonio, Texas; Mrs. M. C. Kersh, Dallas, Texas.

VITAL

FACTS

EVERY

WOMAN

SHOULD

KNOW.

*Teach me the way to a strong body, a pure mind, a noble character.*

*"Ignorance is not innocence."*

THE AMERICAN GYNECOLOGICAL SOCIETY.—The thirty-ninth annual meeting of this society will be held in Boston, Mass., on Tuesday, Wednesday, and Thursday, May 19th, 20th and 21st, with headquarters at the Harvard Club. On Tuesday and Thursday the scientific sessions will be held in the Boston Medical Library Building, and on Wednesday, at the Country Club. The officers of the society are: President, Dr. J. Whitridge Williams, of Baltimore; first vice-president, Dr. Thomas J. Watkins, of Chicago; second vice-president, Dr. Howard C. Taylor; treasurer, Dr. J. Wesley Bovee, of Washington, D. C.; secretary, Dr. Leroy Broun, of New York; other members of the council, Dr. Edward P. Davis, Dr. Reuben Peterson, Dr. Howard A. Kelly, Dr. Henry

C. Coe, Dr. William E. Studdiford, and Dr. Thomas S. Cullen.—*New York Medical Journal.*

